# Valuing reductions in the risk of death in benefit–cost analyses of environment- and climate-health actions

**DOI:** 10.2471/BLT.25.294080

**Published:** 2026-01-22

**Authors:** Frank Pega, Natalie C Momen, Samuel A Agyemang, Laura Bojke, Joan Costa-Font, Laure de Preux, Eli P Fenichel, Bruce Gordon, Martin C Hensher, Richard Johnston, Yuvaraj Krishnamoorthy, Antonios Kolimenakis, Muhammad Ashar Malik, Hiroaki Matsuura, Nhung Nghiem, Bernadette O’Hare, Megha Rathi, Lisa A Robinson, Diarmid Campbell-Lendrum

**Affiliations:** a Department of Environment, Climate Change, One Health and Migration, World Health Organization, Avenue Appia 20, 1211 Geneva 27, Switzerland.; b School of Public Health, University of Ghana, Legon, Ghana.; c Centre for Health Economics, University of York, York, England.; d Department of Health Policy, London School of Economics and Political Science, London, England.; e Department of Economics and Public Policy, Imperial Business School, Imperial College London, London, England.; f Yale School of the Environment, Yale University, New Haven, United States of America (USA).; g Menzies Institute for Medical Research, University of Tasmania, Hobart, Australia.; h Department of Community Medicine, ESIC Medical College & Hospital, Chennai, India.; i Faculty of Arts and Sciences, Aga Khan University, Karachi, Pakistan.; j Faculty of Tourism, Media, and Cultural Studies, Shoin University, Kanagawa, Japan.; k John Curtin School of Medical Research, Australian National University, Canberra, Australia.; l School of Medicine, University of St Andrews, St Andrews, Scotland.; m Harvard TH Chan School of Public Health, Harvard University, Boston, USA.

## Abstract

Economic evaluation is key for efficient allocation of resources in health and related sectors. Actions addressing environmental risk factors and climate change can avert millions of deaths annually, yet valuing reductions in the risk of dying is challenging in benefit–cost analyses. We developed an interim statistical protocol to estimate the value per statistical life for World Health Organization (WHO) Member States, building on the 2019 benefit–cost analysis reference case and latest evidence. Using gross national income per capita based on purchasing power parity, we calculated national estimates for 2024 and projected values to 2100. We aggregated these estimates to produce global, regional and country income group averages, and additional sets for sensitivity and scenario analyses, including for alternative climate change scenarios. Our estimates cover 93.8% (182/194) of Member States, representing 98.4% (7.99 billion/8.12 billion) of the global population. The global average value per statistical life in 2024 was 3.76 million international dollars. By 2100, the global average is projected to increase by 159.8% to 9.77 million international dollars. These estimates provide a basis for valuing expected deaths averted by environment- and climate-health interventions, promoting comparability across analyses. Limitations include reliance on extrapolated values and uncertainty in income projections. More research, especially in low- and middle-income countries, is needed. Because value per statistical life estimates depend on income, analysts must supplement benefit–cost analysis with distributional analyses of benefits and costs across populations. Until WHO updates its recommended methods, these interim estimates offer a pragmatic tool for policy analysis.

## Résumé

L’évaluation économique est essentielle pour une allocation efficace des ressources dans la santé et les secteurs connexes. Les mesures de lutte contre les facteurs de risque environnementaux et le changement climatique permettent d’éviter des millions de décès par an, mais il est difficile d’évaluer la réduction du risque de décès dans les analyses coûts-avantages. À cette fin, nous avons élaboré un protocole statistique provisoire pour estimer la valeur de la vie pour les États membres de l’Organisation mondiale de la Santé (OMS), en nous appuyant sur le cas de référence de 2019 de l’analyse coûts-avantages et les dernières données disponibles. En partant du revenu national brut par habitant en parité de pouvoir d’achat, nous avons calculé des estimations nationales pour 2024 et réalisé des projections jusqu’en 2100. Nous avons agrégé ces estimations afin d’obtenir des moyennes mondiales, régionales et par groupe de revenus des pays, ainsi que des ensembles supplémentaires pour les analyses de sensibilité et de scénarios, y compris pour des scénarios alternatifs de changement climatique. Nos estimations couvrent 93,8% des États membres (182 sur 194), représentant 98,4% de la population mondiale (7,99 milliards sur 8,12 milliards). En 2024, la moyenne mondiale de la valeur de la vie était de 3,76 millions de dollars internationaux. D’ici à 2100, elle devrait augmenter de 159,8% pour atteindre 9,77 millions de dollars internationaux. Ces estimations fournissent une base pour évaluer le nombre de décès évités grâce aux mesures associant protection environnementale et santé d'une part et lutte contre le changement climatique et santé d’autre part, ce qui facilite la comparabilité entre les analyses. Elles sont toutefois limitées en raison du recours à des valeurs extrapolées et de l’incertitude des projections de revenus. Des recherches supplémentaires sont nécessaires à cet égard, en particulier dans les pays à revenu faible et intermédiaire. Étant donné que les estimations de la valeur de la vie dépendent du revenu, les analystes doivent compléter l’analyse coûts-avantages par des analyses de la répartition des avantages et des coûts entre les populations. En attendant que l’OMS mette à jour ses méthodes recommandées, ces estimations provisoires constituent un outil pragmatique pour l’analyse de différentes politiques.

## Resumen

La evaluación económica es fundamental para asignar eficientemente los recursos en el sector sanitario y los sectores conexos. Las acciones dirigidas a factores de riesgo ambientales y al cambio climático pueden evitar millones de muertes anuales, pero la valoración de la reducción del riesgo de morir plantea dificultades en los análisis coste-beneficio. Se elaboró un protocolo estadístico provisional para estimar el valor por vida estadística en los Estados Miembros de la Organización Mundial de la Salud (OMS), a partir del caso de referencia de 2019 para análisis coste-beneficio y de la evidencia más reciente. Utilizando el ingreso nacional bruto per cápita basado en la paridad del poder adquisitivo, se calcularon estimaciones nacionales para 2024 y se proyectaron valores hasta 2100. Estas estimaciones se agregaron para obtener promedios mundiales, regionales y por grupo de ingresos de los países, así como conjuntos adicionales para análisis de sensibilidad y de escenarios, incluidos escenarios alternativos de cambio climático. Las estimaciones abarcan el 93,8% (182/194) de los Estados Miembros, que representan el 98,4% (7,99 mil millones/8,12 mil millones) de la población mundial. El valor medio mundial por vida estadística en 2024 fue de 3,76 millones de dólares internacionales. Para 2100, se proyecta que el promedio mundial aumente un 159,8% hasta 9,77 millones de dólares internacionales. Estas estimaciones proporcionan una base para valorar las muertes previstas evitadas mediante intervenciones relacionadas con la salud ambiental y climática, promoviendo la comparabilidad entre análisis. Entre las limitaciones figuran la dependencia de valores extrapolados y la incertidumbre en las proyecciones de ingresos. Se necesita más investigación, especialmente en los países de ingresos bajos y medios. Dado que las estimaciones del valor por vida estadística dependen del ingreso, los analistas deben complementar el análisis coste-beneficio con análisis distributivos de beneficios y costes entre poblaciones. Hasta que la OMS actualice sus métodos recomendados, estas estimaciones provisionales ofrecen una herramienta pragmática para el análisis de políticas.

## ملخص

يُعدّ التقييم الاقتصادي عاملاً أساسيًا للتخصيص الفعال للموارد في قطاع الصحة والقطاعات ذات الصلة. إن الإجراءات التي تعالج عوامل الخطر البيئية وتغير المناخ، يمكنها أن تتجنب ملايين الوفيات سنويًا، إلا أن تقييم معدلات انخفاض خطر الوفاة يُمثّل تحديًا في تحليلات التكلفة والعائد. لقد قمنا بتطوير بروتوكول إحصائي مؤقت لتقدير قيمة كل حياة إحصائية للدول الأعضاء في منظمة الصحة العالمية، بالاستناد إلى الحالة المرجعية لتحليل التكلفة والعائد لعام 2019 وأحدث الأدلة. وباستخدام إجمالي الدخل القومي للفرد على أساس تعادل القوة الشرائية، قم باحتساب التقديرات الوطنية لعام 2024 وتوقعنا القيم حتى عام 2100. وقمنا بتجميع هذه التقديرات لإنتاج متوسطات الدخل العالمية والإقليمية والقطرية، ومجموعات إضافية لتحليلات الحساسية والسيناريوهات، بما في ذلك سيناريوهات بديلة لتغير المناخ. وتغطي تقديراتنا %93.8 (182/194) من الدول الأعضاء، وهو ما يمثل %98.4 (7.99 مليار/8.12 مليار) من سكان العالم. بلغ متوسط القيمة العالمية لكل حياة إحصائية في عام 2024 نحو 3.76 مليون دولار دولي. بحلول عام 2100، من المتوقع أن يرتفع هذا المتوسط العالمي بنسبة %159.8 ليصل إلى 9.77 مليون دولار دولي. توفر هذه التقديرات أساسًا لتقييم الوفيات المتوقعة التي يمكن تجنبها بفضل التدخلات البيئية والمناخية والصحية، مما يعزز قابلية المقارنة بين التحليلات. تشمل القيود الاعتماد على القيم المستنبطة وعدم اليقين في توقعات الدخل. هناك حاجة إلى مزيد من الأبحاث، لا سيما في الدول ذات الدخل المنخفض والمتوسط. ولأن تقديرات القيمة لكل حياة إحصائية تعتمد على الدخل، يجب على المحللين استكمال تحليل التكلفة والعائد بتحليلات توزيعية للفوائد والتكاليف بين السكان. وحتى تقوم منظمة الصحة العالمية بتحديث طرقها الموصى بها، تُقدم هذه التقديرات المؤقتة أداة عملية لتحليل السياسات.

## 摘要

经济评估是确保在卫生行业和相关行业中实现资源有效分配的重要手段。通过针对环境风险因素和气候变化采取应对措施，每年能够避免数百万人死亡，然而，利用效益成本分析仍难以评估降低死亡风险的价值。根据 2019 年的效益成本分析参考案例和最新证据，我们制定了临时统计方案以估算世卫组织 (WHO) 成员国每个统计生命的价值。利用基于购买力平价算得的人均国民总收入，我们计算了 2024 年各国的估算价值和 2100 年各国的预计价值。我们汇总了这些估算价值以生成全球、区域和国家收入群体的平均价值，并另外构建了多组用于敏感性和情景分析（包括针对不同气候变化情景）的数据。我们计算的估算价值涵盖 93.8% (182/194) 的成员国，覆盖全球 98.4%（79.9 亿/81.2 亿）的人口。2024 年全球每个统计生命的平均价值为 376 万国际元。截至 2100 年，全球平均价值预计会上涨 159.8%，达到 977 万国际元。这些估算价值为通过环境和气候健康干预措施规避的预计死亡人数的价值评估提供了基础，有助于提升不同分析之间的可比性。该方案的局限性包括依赖于外推值及预计收入的不确定性。因此，还需开展更多研究，特别是在中低收入国家。由于每个统计生命的估算价值取决于收入，分析员必须在效益成本分析的基础上辅以不同人群之间的分配式效益成本分析。在世卫组织尚未更新其推荐使用方法之前，这些临时估算价值可被视为适用于开展政策分析的实用工具。

## Резюме

Экономическая оценка является ключевым фактором эффективного распределения ресурсов в здравоохранении и смежных областях. Меры, направленные на снижение воздействия экологических факторов риска и противодействие изменению климата, способны ежегодно предотвращать миллионы смертей, однако оценка снижения риска смерти представляет собой сложную задачу в анализе выгод и затрат. Авторы разработали промежуточный статистический протокол для оценки стоимости одной статистической жизни для государств-членов Всемирной организации здравоохранения (ВОЗ), опираясь на эталонный пример анализа выгод и затрат 2019 года и новейшие научные данные. На основе валового национального дохода на душу населения с учетом паритета покупательной способности были рассчитаны национальные оценки на 2024 год и выполнено прогнозирование значений до 2100 года. Эти оценки были агрегированы для получения глобальных, региональных и средних значений по группам стран с разным уровнем дохода, а также дополнительных наборов данных для анализов чувствительности и сценарных анализов, включая альтернативные сценарии изменения климата. Наши оценки охватывают 93,8% (182 из 194) государств-членов, представляющих 98,4% (7,99 из 8,12 млрд) населения мира. Глобальное среднее значение стоимости одной статистической жизни в 2024 году составило 3,76 млн международных долларов. К 2100 году прогнозируется рост глобального среднего значения на 159,8% – до 9,77 млн международных долларов. Эти данные создают основу для оценки ожидаемого количества предотвращенных смертей в результате экологических и климатических мер в сфере охраны здоровья и способствуют сопоставимости результатов различных анализов. К ограничениям относятся использование экстраполированных значений и неопределенность прогнозов доходов. Необходимы дальнейшие исследования, особенно в странах с низким и средним уровнем дохода. Поскольку оценки стоимости одной статистической жизни зависят от уровня дохода, аналитикам следует дополнять анализ выгод и затрат распределительными анализами выгод и издержек среди различных групп населения. До обновления ВОЗ рекомендуемых методологических подходов эти промежуточные оценки представляют собой прагматичный инструмент для политико-аналитических целей.

## Introduction

Economic evaluation, such as comparing an intervention’s benefits to its costs through benefit–cost analysis (also called cost–benefit analysis), is key for ensuring efficient allocation of scarce resources.^1^ Governments and international organizations, including the World Health Organization (WHO), commonly use benefit–cost analysis to assess non-health sector interventions that affect public health,^2^^–^^5^ such as environment- and climate-health actions. For health sector interventions, WHO recommends cost–effectiveness analysis as a minimum standard.^6^

One in every four deaths worldwide is attributable to environmental risk factors and climate change.^7^ Implementing actions that avert deaths and illnesses caused by these factors will improve social welfare, including protecting human health and well-being. Such actions include environment-health actions that address environmental risk factors, such as air quality, chemicals, occupational hazards, radiation, water, waste, sanitation and hygiene;^8^ and climate-health actions that contribute to climate change adaptation and mitigation, such as building climate-resilient health systems and promoting lower-carbon health systems and societies.^9^ WHO recently estimated that scaling up just five environment- and climate-health actions would avert almost 2 million expected deaths annually.^2^ Methods^10^ and evidence^11^ are improving for quantifying such actions’ health effects, including deaths averted. 

Health policy analysts commonly use benefit–cost analysis and values of reduction in risk of death to economically evaluate actions that avert deaths and illness caused by environmental risk factors and climate change.^12^ Examples include WHO’s benefit–cost analyses of cleaner household energy sources^4^ and Nationally Determined Contributions under the Paris Agreement.^5^ However, a major challenge when conducting benefit–cost analyses is valuing outcomes with insufficient or unavailable market prices, such as reductions in the risk of death. Because reductions in risk of death account for most of the benefits of many policies, applying a theory- and evidence-based approach to estimate their value is crucial. However, a systematic review reported that differences in methodological approach for estimating the value of reductions in risk of death led to widely varying estimates across 120 included studies.^13^ Such methodological variation can affect policy impact assessments. Furthermore, a unique challenge when evaluating environment- and climate-health actions is that benefits and costs accrue over long time horizons and depend on the assumed climate change scenario. The greatest benefits of many climate change mitigation policies will only manifest many years in the future.^11^ Environmental risk factors and climate change are also likely to alter interventions’ health impacts over time, affecting baseline conditions and intervention effectiveness. To address this challenge, the Intergovernmental Panel on Climate Change assesses climate change scenarios of projected socioeconomic changes using the shared socioeconomic pathways of the International Institute for Applied Systems Analysis (Box 1).^19^

Box 1 **Summary of the shared socioeconomic pathways narratives^19^**

***Pathway 1 - Sustainability: taking the green road***

“The world shifts gradually, but pervasively, toward a more sustainable path, emphasizing more inclusive development that respects perceived environmental boundaries.” There are low challenges to mitigation and adaptation to climate change.

***Pathway 2 - Middle of the road***

“The world follows a path in which social, economic, and technological trends do not shift markedly from historical patterns.” There are medium challenges to mitigation and adaptation to climate change.

***Pathway 3 - Regional rivalry: a rocky road***

“A resurgent nationalism, concerns about competitiveness and security, and regional conflicts push countries to increasingly focus on domestic or, at most, regional issues.” There are high challenges to mitigation and adaptation to climate change.

***Pathway 4 - Inequality: a road divided***

“Highly unequal investments in human capital, combined with increasing disparities in economic opportunity and political power, lead to increasing inequalities and stratification both across and within countries.” There are low challenges to mitigation, high challenges to adaptation to climate change.

***Pathway 5 - Fossil-fuelled development: taking the highway***

“This world places increasing faith in competitive markets, innovation and participatory societies to produce rapid technological progress and development of human capital as the path to sustainable development.” There are high challenges to mitigation, low challenges to adaptation to climate change.

Note: full information for each pathway is available in Riahi et al., 2017.^19^

As countries and WHO have committed to conducting more such evaluations,^20^ health policy analysts and practitioners will require accessible tools, including interim estimates, to value mortality reductions in environment- and climate-related health benefit–cost analyses. To address the above challenges, we developed a consistent estimation approach and estimates for valuing mortality reductions, building on the 2019 benefit–cost analysis reference case.^1^

Here, we present our approach and population average estimates of the value of reductions in the risk of death under all these shared socioeconomic pathways, for the world, the six WHO regions, and the four World Bank income groups^18^ for the years 2024–2100. These estimates are intended for use in global and regional benefit–cost analyses that value expected deaths averted by environment- and climate-health actions. Our work provides a consistent, long-time series of such economic estimates under alternative climate change scenarios.

### Value of reductions in the risk of death

Within the benefit–cost analysis conceptual framework, benefits and costs can be valued based on individuals’ willingness to exchange their income for outcomes they experience. Typically, individuals affected by a policy accrue a very small risk reduction, for example a one-in-10 000 change in the risk of dying in the current year. The value of these risk reductions includes pecuniary effects (for example, delaying the costs associated with dying and increasing earnings) and less tangible, non-pecuniary effects (for example, the joys of life).

Fully valuing reductions in the risk of dying requires going beyond pecuniary measures to estimate individual preferences.^13^^,^^21^^–^^25^ The human capital approach,^26^ a market measure sometimes used to value reductions in the risk of dying, solely considers lost production, thereby ignoring the less tangible longevity effects.^1^ These intangible effects can be estimated using survey data on willingness to pay to assess individuals’ stated preferences, or by observing behaviour or using prices of related market goods to assess individuals’ revealed preferences.

Estimates of individual willingness to pay are typically converted into an estimate of the value per expected death averted, by dividing willingness to pay by risk change, resulting in the value per statistical life^27^ or value of prevented fatality.^28^ These values are sometimes referred to as social values, summing the average value for a small risk change across the population. Because value per statistical life is often misinterpreted as the value of saving an individual’s life,^29^ some experts have suggested alternative terms pointing to reduced risk of dying, such as “value of reduced mortality risk.”^30^ The terms “micro-mort”^31^ and “value of standardized mortality unit”^32^ have been used to refer to risk changes values of one-in-1 million and one-in-10 000, respectively. To account for differences in age and life expectancy, the value per statistical life can be converted to estimates for a statistical life year and a quality-adjusted life year.^33^ These life-year values are based on restrictive assumptions that may not hold in practice,^33^ and recommendations on the values’ application vary.^1^

## Developing a new approach

To create a consistent estimation approach for valuing mortality reductions, we first developed an interim statistical protocol. We call it interim because we expect to refine it as new research and data become available. Using this protocol, we calculated national estimates of the value per statistical life for 2024 and projected country-specific values for the years 2025–2100. We then derived global and regional value estimates for the common currency year 2024 and future years 2025–2100 under shared socioeconomic pathway 2. We also produced alternative sets of estimates for use in sensitivity and scenario analyses, including under the other shared socioeconomic pathways (pathways 1, 3, 4 and 5). Below, we describe our approach in more detail. 

### Step 1

To develop our interim statistical protocol, we reviewed previous approaches, guidelines and practices for estimating, applying and reporting the value per statistical life.^1^^–^^5^^,^^15^^,^^23^^,^^24^^,^^34^^–^^36^

### Step 2

To develop global and regional estimates of the value per statistical life, we started to calculate national estimates. Using Equation 1 and the data sources in Table 1, we calculated the value per statistical life (*V*) for each WHO Member State for 2024.
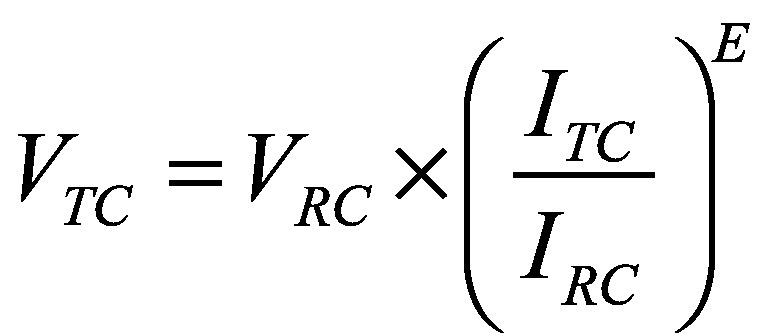
(1)where *TC* is the target country, *RC* is the reference country, *I* is the gross national income (GNI) per capita based on purchasing power parity (PPP) and *E* is the elasticity. 

**Table 1:** Input variables with reference values and sources for extrapolation from reference country (USA) to target country, for the year 2024

Input variable	Value or data extraction and dealing with missing data	Source	Missing data for no. of WHO Member States and % of the 2024 global population^14^
**Value per statistical life_reference country_** **(*V_RC_* in Equation 1)**	For 2024, Int$ 13 429 139^a^	United States Department of Health and Human Services^36^	NA
**GNI per capita, PPP_reference country_** **(*I_RC_*)**	For 2024, Int$ 85 980	World Bank^37^	NA
**GNI per capita, PPP_target country_** **(*V_TC_*)**	We extracted from the source the latest available data point for the years 2022–2024 for the 185 WHO Member States with available data. The most recent available GNI per capita, PPP from these three years was used. For Member States without GNI per capita, PPP, reported in the source for any of the years 2022–2024, we set the value per statistical life to missing	World Bank^37^	Nine WHO Member States^b^ representing 1.5% of global population
**Elasticity** **(*E*)**	Primary analysis: we applied a value per statistical life income elasticity of 1 to all target countries. Sensitivity analysis for value per statistical life income elasticities: we applied varying value per statistical life income elasticities dependent on World Bank income group classification for the 2024 currency year extracted from the source.^c^ For WHO Member States that are unclassified, we set the value per statistical life to missing	Sensitivity analysis: World Bank^18^	Primary analysis: Zero WHO Member States representing 0.0% of global population Sensitivity analysis: Four WHO Member States^d^ representing 2.0% of global population

GNI: gross national income; Int$: international dollars; NA: not applicable; PPP: purchasing power parity; WHO: World Health Organization.

^a^ Reported by the United States Department of Health and Human Services as 13 429 139 United States dollars,^36^ which for the USA is the same value in international dollars.

^b^ Cook Islands, Cuba, Democratic People's Republic of Korea, Eritrea, Monaco, Niue, South Sudan, Bolivarian Republic of Venezuela and Yemen.

^c^ The classification for the Fiscal Year 2026 is based on 2024 data.^18^

^d^ Cook Islands, Ethiopia, Niue and Bolivarian Republic of Venezuela.

We selected the United States of America as the reference country, as it has the most studies estimating value per statistical life and is often used as the starting point for value per statistical life extrapolations.^1^^,^^13^^,^^15^ Several reviews have reached broadly consistent estimates for the USA, after adjusting for inflation and real income growth to the same reference year. These include reviews reflected in guidance followed by the United States Department of Health and Human Services,^36^ Department of Transportation,^35^ and Environmental Protection Agency,^34^ and in bias-corrected estimates.^38^^,^^39^ Because the Department of Health and Human Services conducted the most recent USA federal agency review, we used its value per statistical life as the reference for extrapolation.

We started with a reference ratio of 156 for 2024, calculated by dividing the USA value per statistical life (13 429 139 international dollars, Int$) by its GNI per capita based on PPP (Int$ 85 980). We measure income of target countries using 2024 GNI per capita, PPP, sourced from the World Bank income level data released in 2025, which is consistently estimated and widely used (Table 1).

Adjusting values across countries requires applying the income elasticity of the value per statistical life (Equation 1), which estimates how the value per statistical life changes as income changes. We used an elasticity of 1.0 for all countries in our primary analysis, based on systematic reviews and meta-analyses,^15^^,^^21^^–^^24^ aligned with recent best practice guidelines,^25^ and consistent with several recent global analyses.^2^^,^^15^^,^^40^^–^^43^ Because value per statistical life estimates are available primarily from high- and upper-middle income countries, the appropriate income elasticity for extrapolation becomes more uncertain as income levels decrease.

### Step 3

To estimate each country’s value per statistical life for 2025–2100, we used the estimated national values per statistical life (*V*) for 2024 and the projected national GNI per capita, PPP (*I*), for 2024–2100. We populated Equation 2 with data from sources presented in Table 2 for each country:
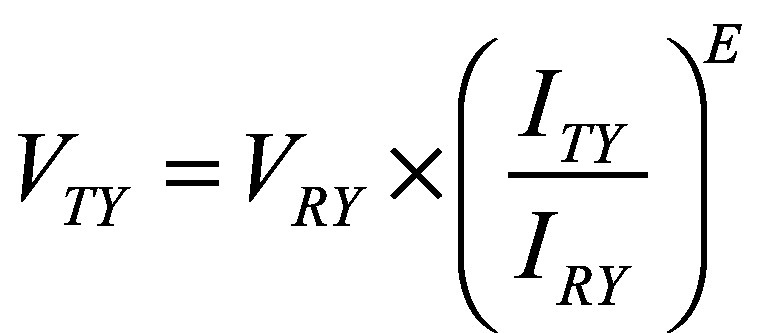
(2)where *TY* is the target year and *RY* is the reference year 2024. 

**Table 2:** Input variables with reference values and sources for extrapolation from reference year (2024) to target future year

Input variable	Value or data extraction, and dealing with missing data	Source	Data missing for no. of WHO Member States and % of the 2024 global population^14^
**Value per statistical life_reference year_** **(*V_RY_* in Equation 2)**	We used our value per statistical life estimates for 2024 as calculated for the 185 WHO Member States in Step 2. For the nine WHO Member States that a 2024 value per statistical life could not be calculated, we set values to missing	Output from Step 2 (Equation 1)	Nine WHO Member States^a^ representing 1.5% of global population
**GNI per capita, PPP_reference year_** **(*I_RY_*)**	We extracted real income, measured as GNI per capita, PPP, for 2024 from the source for the 185 Member States in Step 2 with available 2024 value per statistical life estimates	World Bank^15^	Nine WHO Member States^a^ representing 1.5% of global population
**GNI per capita, PPP_target year_** **(*I_TY_*)**	We calculated real income, measured as GNI per capita, PPP, in the target future year (e.g. 2025) for each target country, using data sets 1–4 and the methods described below, for 182 WHO Member States and for each shared socioeconomic pathway	(see below)	Twelve WHO Member States^b^ representing 1.5% of global population
Data set 1: GNI, PPP, for the year 2024	We extracted the latest available data point for GNI, PPP, from the source for 185 WHO Member States for the years 2021–2024. If a Member State had no data point for any of the years 2021–2024, we set the value as missing	World Bank^16^	Nine WHO Member States^a^ representing 1.5% of global population
Data set 2: compound annual growth rates for GDP for the years 2024–2100	Ideally, in extrapolation of GNI per capita, PPP, we would use the same income measure. However, projections of changes in real GNI per capita, PPP, are not available for all Member States and all years. Therefore, we assumed that the rate of change in GNI per capita, PPP, is the same as the rate of change in GDP per capita, calculated by dividing projected GDP for each country by its population in each target year. These GDP values were unadjusted for inflation, reflected only changes in real value and expressed in 2024 Int$. We report them as: 2024 Int$, 2025 income level. We extracted GDP projections from the source for the years 2025–2100. As the GDP projections were reported in five-year increments, we calculated the compound annual growth rate for each year within each increment. We then applied these rates to the latest data point on GNI, PPP, over 2021–2024 (data set 1) to estimate GNI, PPP, for each of the years 2025–2100 under shared socioeconomic pathway 2. As the projections were not available for 2025, we applied the compound annual growth rate for 2026–2030 to that year. For WHO Member States without national GDP projections, we assigned the GDP projections for the country groupings in which the countries are included^17^^,c^	International Institute for Applied Systems Analysis^17^^,d^	Four WHO Member States^f^ representing < 0.1% of global population
Data set 3: population estimates for the year 2023	We extracted the population estimate for 2023 from the Estimates, 1950–2023 in the source	UN^14^	Zero WHO Member States representing 0.0% of global population
Data set 4: compound annual growth rates for the population for the years 2023–2100	We extracted the population projections for the years 2020–2100 for the shared socioeconomic pathway 2 scenario from the source. The model for these population projections is internally consistent with the model used for GDP projections in data set 2. As the population projections are reported in five-year increments, we calculated the compound annual growth rate for each year within each increment. We then applied the rates to the UN population estimates for 2023 (data set 3) to estimate the population for each of the years 2024–2100 under the shared socioeconomic pathway 2. Finally, for each estimation year, we divided the projected GNI, PPP, by the projected population to produce projected GNI per capita, PPP. For WHO Member States without population projections, we assign the population projections for the country groupings in which the countries are included.^17^^,e^ While we chose the shared socioeconomic pathway 2 scenario for primary analyses, we also produced alternative estimates sets for the other four shared socioeconomic pathway scenarios (Box 1), using the GDP projections (data set 2) and population projections (data set 4) for these pathways	International Institute for Applied Systems Analysis^17^	Four WHO Member States^f^ representing < 0.1% of global population
**Elasticity** **(*E*)**	Primary analyses: we applied a value per statistical life income elasticity of 1 to all target countries. Sensitivity analyses: we applied varying value per statistical life income elasticities dependent on World Bank income group classification for the common currency year (2024, extracted from the classification for the fiscal year 2026 in the source, which is based on 2024 data). For the unclassified WHO Member States for fiscal year 2026, no value per statistical life was calculated for the value per statistical life income elasticities sensitivity analysis, and the value was set to missing	Sensitivity analysis: World Bank^18^	Primary analysis: zero WHO Member States representing 0.0% of global population Sensitivity analysis: four WHO Member States^g^ representing 2.0% of global population

GDP: gross domestic product; GNI: gross national income; Int$: international dollars; PPP: purchasing power parity; UN: United Nations; WHO: World Health Organization.

^a^ Cook Islands, Cuba, Democratic People's Republic of Korea, Eritrea, Monaco, Niue, South Sudan, Bolivarian Republic of Venezuela and Yemen.

^b^ Andorra, Cook Islands, Cuba, Democratic People's Republic of Korea, Dominica, Eritrea, Monaco, Niue, San Marino, South Sudan, Bolivarian Republic of Venezuela and Yemen.

^c^ For WHO Member States, for which national GDP projections were unavailable from International Institute for Applied Systems Analysis 2024,^17^ but for which GDP projections were available from this source for country groupings that included the country, we assigned the country grouping’s GDP projection to the country. For Eritrea, Libya, Somalia and South Sudan, we assigned the projection for the country grouping called Africa (R10); for Democratic People's Republic of Korea, we assigned the country grouping China^+^ (R10); for Afghanistan, we assigned the country grouping India+ (R10); for Cuba, Guyana, St Kitts and Nevis, and Bolivarian Republic of Venezuela, we assigned the country grouping Latin America (R10); for Syrian Arab Republic, we assigned the country grouping Middle East (R10); and for Cook Islands, Federated States of Micronesia, Kiribati, Marshall Islands, Nauru, Palau, Papua New Guinea, Solomon Islands, Timor-Leste, Tonga, Tuvalu, Vanuatu and Samoa, we assigned the country grouping Rest of Asia (R10). The WHO Member States that were assigned the projection for a country grouping represent 2.3% of the 2024 global population.^14^

^d^ We extracted projections from the GDP 2023 model for shared socioeconomic pathway 2.^17^

^e^ For WHO Member States, for which national population projections were unavailable from International Institute for Applied Systems Analysis 2024^17^, but for which population projections were available from the source for country groupings that included the country, we assigned the country grouping’s population projection to the country. For St Kitts and Nevis, we assigned the country grouping called Latin America (R10), and for Cook Islands, Marshall Islands, Nauru, Niue, Palau and Tuvalu, we assigned the country grouping Rest of Asia (R10). The WHO Member States that were assigned a country grouping’s projection represent < 0.1% of the 2024 global population.^14^

^f^ Andorra, Dominica, Monaco, and San Marino.

^g^ Cook Islands, Ethiopia, Niue, and Bolivarian Republic of Venezuela.

### Step 4

To produce primary global, regional and income group value per statistical life estimates for 2024–2100, we combined national estimates to produce average values per statistical life globally and for the six WHO regions (African Region, Region of the Americas, Eastern Mediterranean Region, European Region, South-East Asia Region, Western Pacific Region) and for four income groups (low-income, lower-middle income, upper-middle income, high-income groups). To estimate these values, we calculated each country’s population (*Pop_Cx_*) as a proportion (*P_Cx_*) of the respective group population (*P_G_*; Equation 3),^14^ applied this proportion to the country’s value per statistical life (*V_Cx_*) to produce a population-weighted value per statistical life (*PopwV*; Equation 4), and summed these population-weighted values.
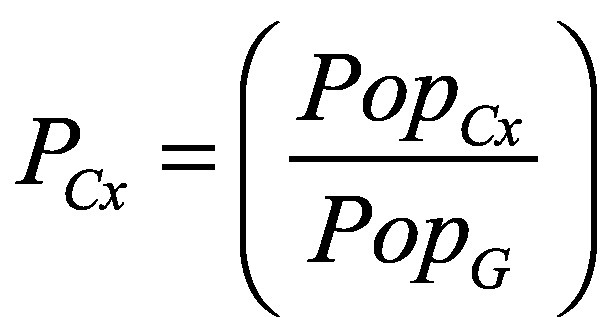
(3)

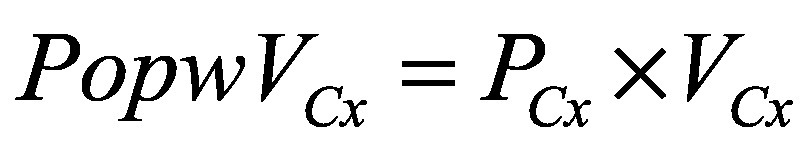
(4)Additionally, we produced average values per statistical life for Least Developed Countries and Small Island Developing States. The online repository contains country lists for each category.^44^

### Step 5

We produced three additional sets of value per statistical life estimates for use in sensitivity and scenario analyses. First, we produced high and low value per statistical life estimates, starting with the United States Department of Health and Human Services’ reasonable high and low estimates.^36^

Second, we varied the value per statistical life elasticity by income group for the fiscal year 2026:^18^ 1.5 for low-income countries, based on comparison of the USA estimate to estimates from the few studies conducted in low-income countries;^1^ 1.2 for lower- and upper-middle income countries, based on expert judgement; and 1.0 for high-income countries. Although extrapolating from previous studies has limitations, these elasticities seem reasonable, since we expect willingness to pay for small reductions in the risk of death to decline as income decreases. If this approach yielded a target country value of < 20 times GNI per capita, PPP, then we used 20 times GNI per capita, PPP, as a proxy for future income, because the value per statistical life is expected to exceed future lifetime income.

Third, we produced estimates of a value per statistical life for the four other shared socioeconomic pathways (pathways 1, 3, 4 and 5; Box 1 and Table 2).^19^

## Interim estimates

Using our interim approach, we estimated value per statistical life for 93.8% (182/194) of WHO Member States, capturing 98.4% (7.99 billion/8.12 billion) of the 2024 global population.^14^ For the remaining 6.2% (12/194) of these countries, value per statistical life could not be estimated due to missing data (Table 1 and Table 2). For countries missing estimates, the value per statistical life is not zero and could be proxied using WHO region or income group estimates.

**Fig 1:**
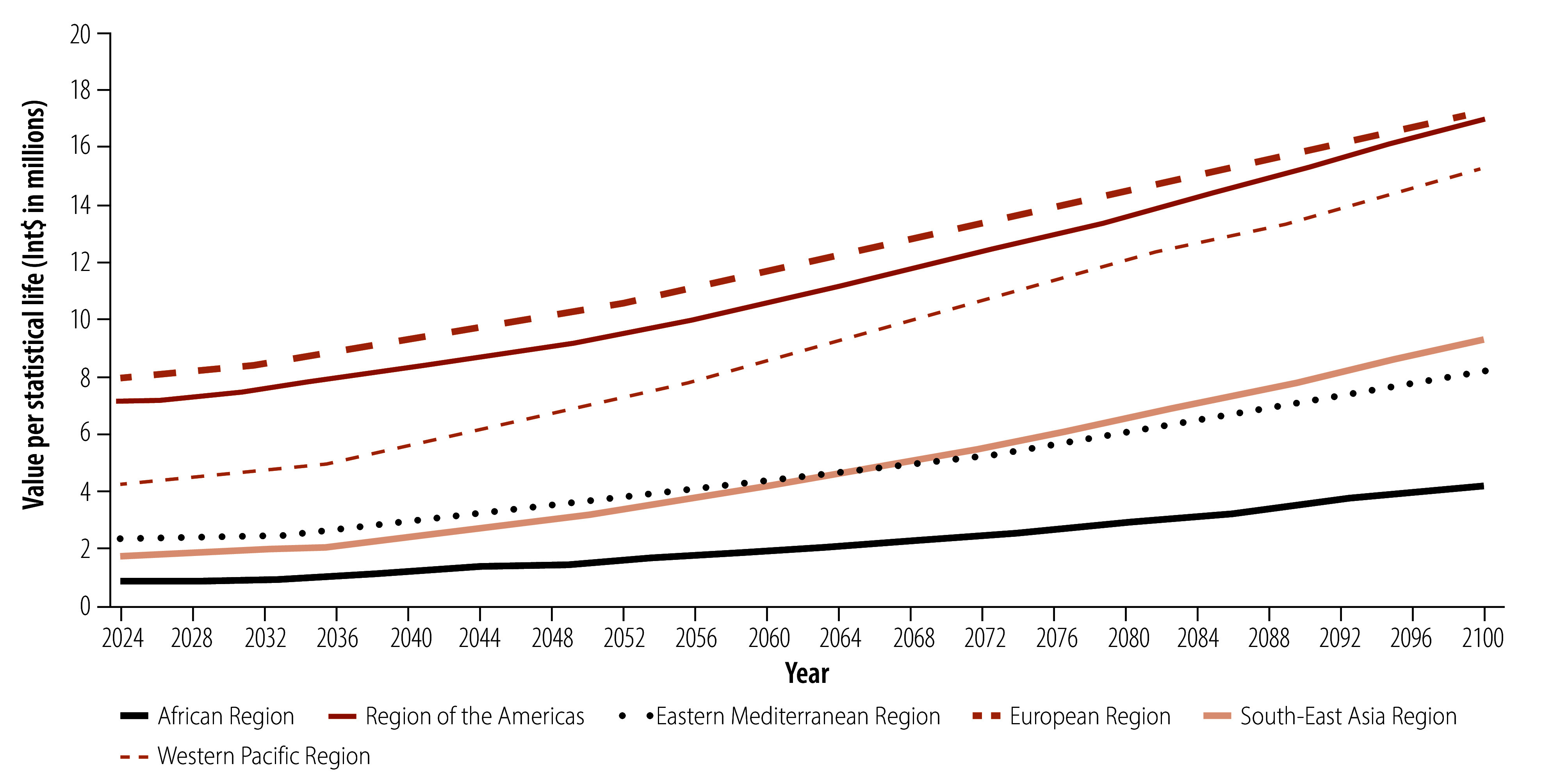
Estimates of value per statistical life, by WHO region, 182 countries, 2024–2100

The estimated global average value in 2024 was Int$ 3.76 million. The estimated averages for regions and income groups ranged from Int$ 0.85 to Int$ 8.00 million and Int$ 0.36 to Int$ 10.29 million, respectively. For Least Developed Countries and Small Island Developing States, the estimates for 2024 were Int$ 0.70 million and Int$ 3.87 million, respectively (Fig. 1 and Table 3). All estimates for all groups under all shared socioeconomic pathways are available in the online repository.^44^

Int$: international dollars; WHO: World Health Organization.

Notes: estimates are 2024 Int$ and we used the income level data released in 2025. We did the primary analysis using the shared socioeconomic pathway 2 scenario (Box 1).

**Table 3:** Global, regional and income group estimates for value per statistical life, 182 countries, 2024–2100

Group	Estimate of value per statistical life, 2024 Int$ in millions,^a^ by year																
	2024	2025	2030	2035	2040	2045	2050	2055	2060	2065	2070	2075	2080	2085	2090	2095	2100
**Global**	3.76	3.77	3.89	4.13	4.39	4.74	5.13	5.53	5.91	6.36	6.83	7.30	7.76	8.23	8.72	9.25	9.77
**WHO Region**																	
African Region	0.85	0.86	0.90	1.02	1.16	1.31	1.47	1.66	1.86	2.08	2.32	2.58	2.86	3.16	3.50	3.86	4.24
Region of the Americas	7.12	7.13	7.43	7.84	8.29	8.79	9.35	9.98	10.63	11.33	12.07	12.82	13.60	14.42	15.28	16.16	17.03
Eastern Mediterranean Region	2.31	2.34	2.39	2.60	2.85	3.12	3.42	3.77	4.16	4.59	5.06	5.54	6.03	6.52	7.04	7.59	8.14
European Region	7.99	8.04	8.43	8.95	9.45	10.00	10.55	11.12	11.70	12.38	13.06	13.68	14.31	14.98	15.70	16.47	17.27
South-East Asia Region	1.76	1.77	1.85	2.10	2.40	2.77	3.18	3.63	4.11	4.65	5.24	5.87	6.54	7.21	7.91	8.61	9.32
Western Pacific Region	4.24	4.27	4.58	4.99	5.41	6.12	6.94	7.76	8.46	9.38	10.34	11.24	12.04	12.80	13.58	14.45	15.23
**World Bank income group** ^18^																	
Low income	0.36	0.36	0.37	0.45	0.53	0.63	0.74	0.86	1.00	1.15	1.32	1.52	1.73	1.97	2.24	2.53	2.84
Lower-middle income	1.55	1.56	1.63	1.84	2.10	2.39	2.72	3.07	3.46	3.89	4.34	4.82	5.33	5.84	6.37	6.92	7.47
Upper-middle income	3.82	3.81	3.94	4.33	4.79	5.35	6.07	6.78	7.47	8.24	9.04	9.78	10.44	11.05	11.67	12.34	12.95
High income	10.29	10.43	11.23	11.85	12.37	13.21	13.91	14.68	15.35	16.28	17.22	18.10	19.00	19.95	20.96	21.99	23.01
**Other classifications**																	
Least Developed Countries	0.70	0.70	0.70	0.78	0.88	0.99	1.12	1.26	1.43	1.61	1.82	2.05	2.31	2.59	2.90	3.22	3.57
Small Island Developing States	3.87	3.87	3.65	3.93	4.27	4.60	4.96	5.28	5.57	5.87	6.17	6.42	6.65	6.87	7.09	7.34	7.61

Int$: international dollars; WHO: World Health Organization.

^a^ We used the income level data released in 2025.

Note: due to lack of input data, we could not estimate the value per statistical life for the 2024–2100 period for 12 WHO Member States: Andorra, Cook Islands, Cuba, Democratic People's Republic of Korea, Dominica, Eritrea, Monaco, Niue, San Marino, South Sudan, Bolivarian Republic of Venezuela and Yemen. While these countries represent only 1.5% of the 2024 global population, this omission could lead to some differences in global and regional (and income level) estimates if these countries differ in their willingness to pay.

**Fig. 2:**
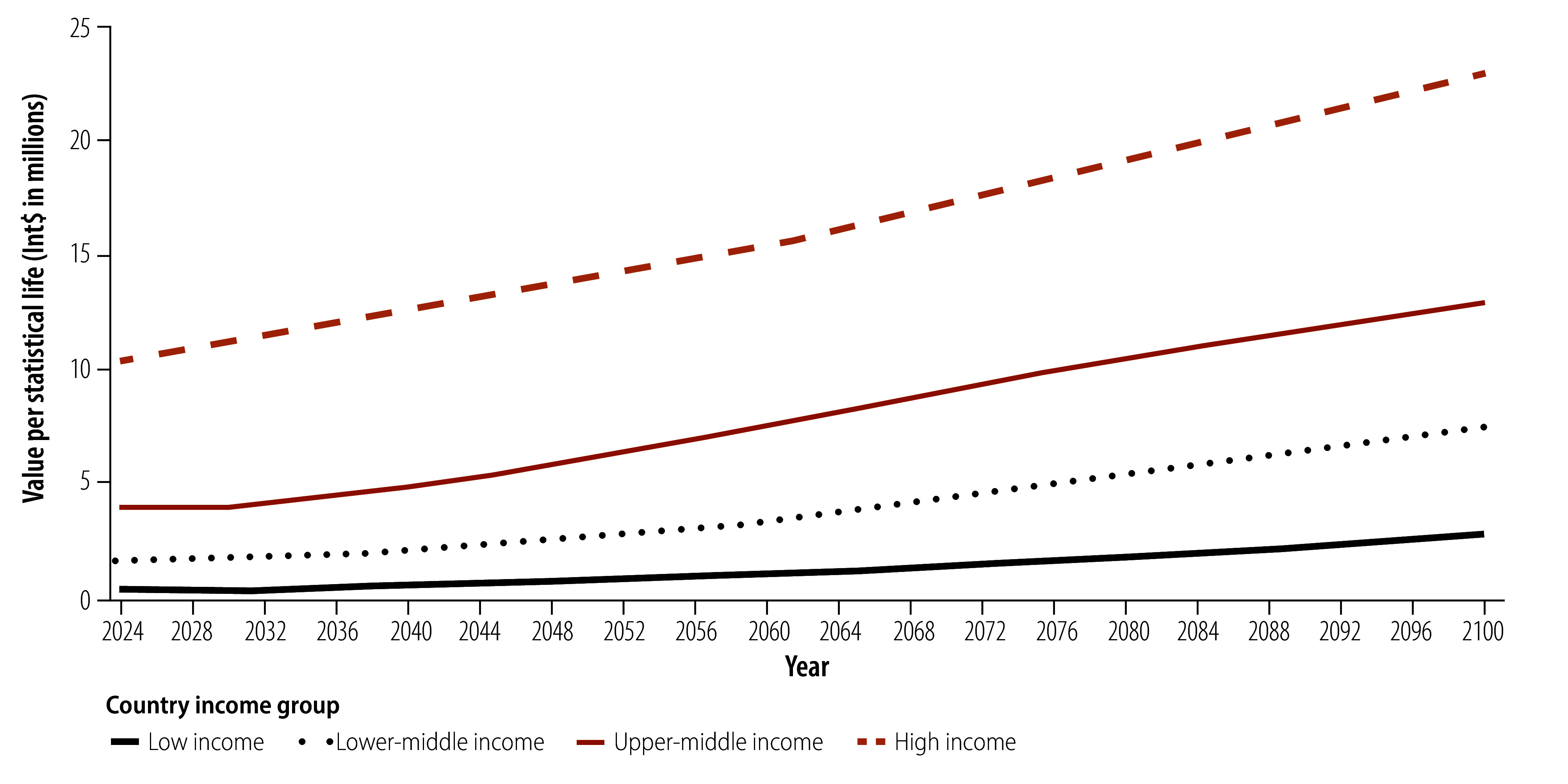
Estimates of value per statistical life, by income group, 182 countries, 2024–2100

**Fig. 3:**
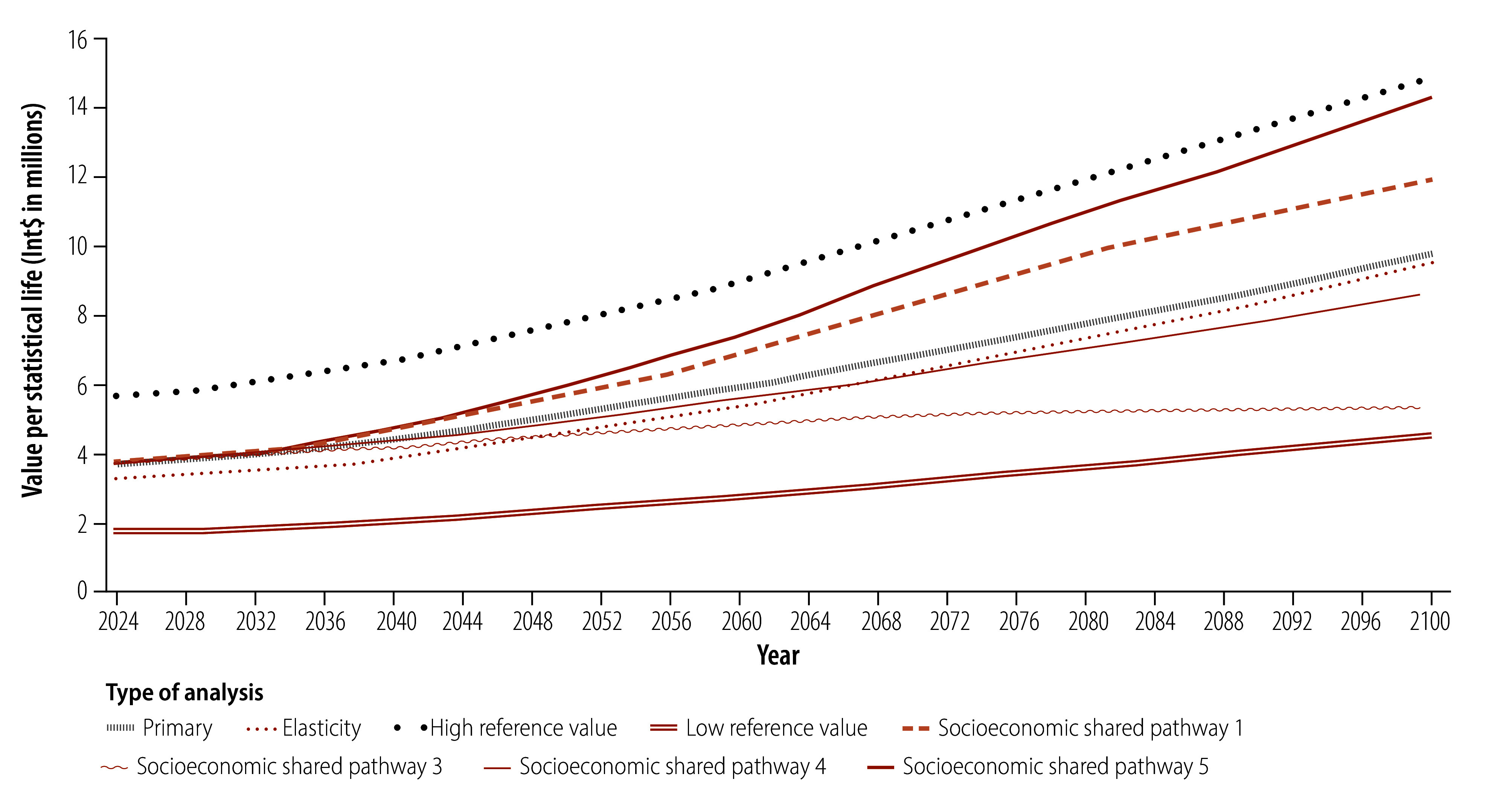
Estimates of value per statistical life, by analysis, 182 countries, 2024–2100

Between 2024 and 2100, the projected global average value per statistical life will increase by 159.8% from Int$ 3.76 to Int$ 9.77 million. Similarly, the value per statistical life is projected to increase in all regions, with the largest increase (429.5%; from Int$ 1.76 million to Int$ 9.32 million) in the South-East Asia Region; and the smallest in the European Region (116.1%; from Int$ 7.99 million to Int$ 17.27 million; Fig. 1). These trends are driven primarily by increases in gross domestic product (GDP) per capita projected under shared socioeconomic pathway 2. The value per statistical life increases for all income groups, most in low-income countries (688.9%; from Int$ 0.36 million to Int$ 2.84 million) and least in high-income countries (123.6%; from Int$ 10.29 million to Int$ 23.01 million; Fig. 2). Fig. 3 shows global estimates over time for alternative shared socioeconomic pathways.

Int$: international dollars.

Notes: estimates are 2024 Int$ and we used the income level data released in 2025. We did the primary analysis using the shared socioeconomic pathway 2 scenario (Box 1).

Int$: international dollars.

Notes: estimates are 2024 Int$ and we used the income level data released in 2025. The analyses for which estimates are presented are the primary analyses and the analyses to produce alternative estimates of value of statistical life as detailed in Step 5.

Table 4 shows the relationship between estimates of the value per statistical life and individual willingness to pay for a one-in-10 000 change in risk, expressed as a percentage of income by income group. The table presents both the primary estimates and those from sensitivity or scenario analyses. 

**Table 4:** Applied income elasticity and estimates of value per statistical life, willingness to pay for a 1-in-10 000 risk change and willingness to pay as percentage of income for different analyses, by country income group, 182 countries, 2024

World Bank income group^18^	Primary analysis					Value per statistical life income elasticities sensitivity analysis			
	Income elasticity	Estimated 2024 value per statistical life, Int$ in millions	Willingness to pay for one-in-10 000 risk change, Int$	Willingness to pay, % of GNI per capita, PPP		Income elasticity	Estimated 2024 value per statistical life, Int$ in millions	Willingness to pay for one-in-10 000 risk change, Int$	Willingness to pay, % of GNI per capita, PPP
**Low income**	1.0	0.36	35.89	1.56		1.50	0.06	6.15	0.27
**Lower-middle income**	1.0	1.55	154.52	1.55		1.20	1.02	101.56	1.02
**Upper-middle income**	1.0	3.82	381.72	1.99		1.20	2.99	298.92	1.56
**High income**	1.0	10.29	1029.21	2.48		1.00	10.29	1029.21	2.48

GNI: Gross national income; Int$: international dollars; PPP: purchasing power parity.

Notes: estimates are 2024 Int$ and we used the income level data released in 2025.

## Strengths and limitations

Our approach builds on reviews of best practice methods and the latest available data sources, extending the benefit–cost analysis reference case^1^ already used by WHO.^4^ Using this approach, we provide comprehensive and consistent estimates of value per statistical life across settings, which analysts can easily use in environment- and climate-health benefit–cost analyses, promoting comparability and easing conduct of such analyses.

However, these estimates have empirical limitations. First, few studies on value per statistical life exist from low- and middle-income countries. For countries classified as low- and lower-middle income in the 2025 fiscal year, 17 value per statistical life studies are available, covering 22 233 participants from three low-income countries and 10 lower-middle income countries in four WHO regions (see online repository).^13^^,^^15^ Estimates of value per statistical life for low- and middle-income countries are therefore often extrapolated from values for high-income countries, using different base estimates and elasticities.^1^^,^^22^^–^^25^ Although more evidence exists for high-income countries, inconsistencies and gaps in primary and synthesized evidence result in uncertainty even for these countries. More research is needed to determine whether these value per statistical life ranges are reasonable when extrapolated to other countries.

Second, our estimates are extrapolated from base values from the United States Department of Health and Human Services.^36^ However, each data source for extrapolation has uncertainty. Most importantly, small changes in income elasticity can lead to very large changes in values, yet limited evidence exists on the most appropriate elasticities to use when producing global estimates, given limited evidence from low- and lower-middle-income countries. The income elasticities we use combine evidence from a USA cohort study^45^ and evidence syntheses,^15^^,^^22^^–^^26^ but ultimately require substantial expert judgement. Because the elasticity depends on the base value per statistical life, starting with a different base may require using a different elasticity. As each individual value per statistical life study has limitations,^46^ our approach relies on evidence syntheses.

Third, when extrapolating across target countries and years, we would ideally use the income measure used in the underlying studies on value per statistical life and adjust for other differences between study and target populations. Studies on value per statistical life typically use estimates of individual or household income.^25^ However, consistently estimated income measures are not available for all countries. We therefore used GNI per capita, PPP, as a proxy, even though there is substantial uncertainty in projecting GNI per capita, PPP, 75 years into the future. We are unaware of projections of changes in real GNI per capita, PPP, for all countries and years included in our estimations. Therefore, we assumed GNI per capita changes like GDP per capita as projected under shared socioeconomic pathways. An alternative approach would be providing estimates for a short time frame only, but this would not provide estimates needed to value long-term impacts of environment- and climate-health actions. Additionally, value per statistical life likely varies due to non-income characteristics, such as sex and age, socioeconomic and cultural contexts and risk of death types; however, their effects on value per statistical life remain uncertain.^15^^,^^23^^,^^24^

Fourth, we do not estimate the value of preventing non-fatal diseases and injuries. Willingness-to-pay studies for non-fatal outcomes are sparse.^1^^,^^47^ Future work should develop estimates of such values for prioritized non-fatal outcomes and determine how to transfer these values across populations. However, because reductions in death risk dominate benefit values for many environment- and climate-health policies, developing a consistent approach for their valuation is an important advance.

Fifth, we did not produce statistical uncertainty ranges, as statistical uncertainty measures were unavailable for the input data. Instead, we provide values for sensitivity analyses to explore uncertainty.

Finally, the concept of value per statistical life applies a normative framework consistent with welfare economic theory underlying benefit–cost analysis.^1^^,^^46^ Value per statistical life is derived from an individual’s preferences for exchanging their income for the risk reductions they would experience. Consequently, value per statistical life varies by income level, yielding smaller values for those on lower incomes, both within and across countries. Therefore, benefit–cost analyses using value per statistical life should be supplemented with distributional analysis of benefits and costs across populations defined by different income levels.^1^^,^^28^^,^^34^^,^^36^ More equity-sensitive concepts and measures have been proposed, but pragmatic methods for implementing these are only emerging.^48^^–^^50^

## Application in policy and practice

We provide estimates for use in regional and global benefit–cost analyses that explore potentially beneficial investments in environment- and climate-health interventions. For analysis of policies implemented at other levels, the appropriate value per statistical life should be used, for example, national value per statistical life for national-level analyses, as can be calculated using Equation 1 and Equation 2.

While we used the shared socioeconomic pathway 2 scenario for the primary analysis, we recognize health policy analysts and practitioners may use different pathways for their primary analyses (online repository).^44^

Conceptually, value per statistical life reflects the value of a shift in the survival curve. Because we will all die eventually, reducing the risk of dying in the current year increases the risk of dying in a future year. Hence these values implicitly reflect how individuals discount the effects of a current risk reduction on risks in future years.^33^ Estimates should be applied to expected deaths averted per year of intervention implementation and then discounted to present values, using the approach and rate used throughout the analysis. We adjusted base estimates for 2024 inflation and follow common practice of not adjusting for future inflation given uncertainties.^1^ For analyses where benefits and costs are estimated using currency years other than 2024, reference value per statistical life and income estimates will need adjusting.

Because estimates of value per statistical life depend on income, analysts must assess the distribution of the action’s benefits and costs across advantaged and disadvantaged populations to inform equity considerations. Methods are emerging for valuing reductions in health inequities, for example distributional weights.^50^

## Next steps

More primary research on the value per statistical life, especially in low- and middle-income countries, is needed to advance our understanding of these values. For example, the 2025 Organisation for Economic Co-operation and Development systematic review on value per statistical life includes data warranting further investigation, including income elasticity data.^15^ As new data and methods become available, the presented interim approach can be updated. The methods presented here should not be considered or cited as WHO’s recommended methods to estimate value per statistical life. However, the methods provide a pragmatic basis to estimate value per statistical life for environment- and climate-health interventions until WHO-recommended methods are developed. WHO is currently updating its recommended methods for economic evaluation of health-improving interventions, including the use of benefit–cost analysis and the value per statistical life. 
